# Silk Microneedles for Targeted Epidermal Delivery of Antifreeze Proteins

**DOI:** 10.1021/acsomega.5c11565

**Published:** 2026-03-16

**Authors:** Brian T. Penney, Antonio Reyes, Calvin L. Jones, Jordan Daw, Amevi Semodji, Konrad Meister, Sophia K. Theodossiou

**Affiliations:** † Biomedical Engineering Doctoral Program, 1791 Boise State University, Boise, Idaho 83725, United States; ‡ Department of Chemistry and Biochemistry, Boise State University, Boise, Idaho 83725, United States; § Department of Mechanical and Biomedical Engineering, Boise State University, 1910 University Dr MS2085, Boise, Idaho 83725, United States; ∥ Max Planck Institute for Polymer Research, Mainz 55128, Germany; ⊥ Spencer Fox Eccles School of Medicine, University of Utah, Salt Lake City, Utah 84113, United States

## Abstract

Antifreeze proteins (AFPs) from cold-adapted organisms excel at modifying ice crystal growth and inhibiting ice recrystallization, making them highly attractive compounds for cryopreservation and frostbite prevention. Traditional drug delivery methods are inadequate for AFPs because their large molecular weight and charge prevent them from crossing cellular membranes and from reaching target tissues through oral, intramuscular, or intravenous routes. Transdermal microneedle (MN) patches offer a promising alternative for delivering AFPs through capillary networks just beneath the epidermis or into specific cellular compartments. However, current materials and protocols are inadequate for encapsulating, storing, and delivering functional AFPs. Here, we demonstrate that silk MN patches, manufactured using a novel heat-free fabrication method, can encapsulate and deliver AFPs into porcine skin, an *ex vivo* model that closely resembles human tissue. We show that the silk MN patches preserve AFP activity, achieve effective penetration and degradation in the tissue, and successfully deliver functional proteins into the porcine epidermis. These results highlight the potential of encapsulating AFPs as a first step toward their use for frostbite prevention and targeted long-term tissue and organ cryostorage applications. Our findings support the use of silk fibroin as a raw material for manufacturing transdermal microneedle patches to deliver emerging therapeutics.

## Graphical abstract

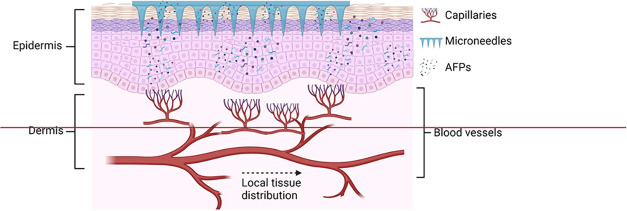

## Introduction

Ice formation presents a major technological challenge, with consequences for settings as diverse as oil pipelines, wind turbines, road surfaces, and frozen food. In biological context, uncontrolled ice crystal growth is especially harmful, causing cellular and tissue damage, and mechanical failures.^1–5^ These effects are especially pronounced during cryopreservation, where ice-induced damage limits the long-term storage of cells and tissues critical to biomedical research. Prolonged exposure to subzero temperatures can also cause severe cold injuries such as frostbite, and can result in tissue necrosis, loss of function, and long-term aesthetic and sensory complications of the affected areas.^6–9^ Cold-induced injuries mostly result from intracellular and extracellular ice formation and become severe when entire tissue layers such as the skin epidermis are affected.^9^ A major limitation in preventing ice-related tissue damage is the challenge of delivering cryoprotectants directly to targeted regions of skin and tissue. Current methods rely on systemic circulation or intravenous delivery, which often result in inadequate local concentrations and unwanted side effects.

Cold-adapted organisms have evolved biomolecular strategies to thrive in subzero temperatures by producing antifreeze proteins (AFPs) and antifreeze glycoproteins (AFGPs). AF­(G)­Ps possess unique properties such as inhibiting ice recrystallization, dynamically shaping ice crystals, and lowering the freezing point of solutions in a noncolligative manner.^10^ AF­(G)­Ps have attracted significant interest as protective agents in cryopreservation and cold-induced injuries.^11–13^ Their beneficial effects have been demonstrated in a range of applications like preventing cold-induced damage in human fibroblasts, preserving fertility in bovine oocytes and the functionality in cryopreserved rat livers. However, some studies also report limited or no protection in complex systems such as cardiac explants, highlighting existing inconsistencies in the usage of AF­(G)­Ps in cryopreservation.^14^ Variable results can stem from protein quality, or the difficulty of delivering AF­(G)­Ps to their tissue targets at effective concentrations and sustained durations. The molecular size and charges of AF­(G)­Ps prevents cellular uptake and has limited their potential for preventing severe frostbite and cold-induced injuries. To reach cells, AF­(G)­Ps may need to be delivered into or near cells by targeting interstitial tissues or localized circulation.

Transdermal microneedle (MN) patches are emerging medical devices capable of delivering therapeutic payloads into the skin by penetrating the stratum corneum and accessing the underlying epidermis, including capillary-rich regions, without stimulating pain receptors.^15–18^ MNs are projected to be a 4.1 billion USD market in the U.S. by 2030, despite all but one product still being in the development and trial stages.^19,20^ Unlike traditional transdermal patches that rely on passive diffusion, MNs create micron-scale conduits that enable efficient, localized delivery.^21^ The composition and fabrication methods of MNs can be tailored to accommodate diverse therapeutic agents, making them a promising platform for addressing the challenges associated with AFP delivery. Fabrication methods that eliminate steps requiring temperatures above 35 °C or below 4 °C are of particular interest, as numerous potential drugs or other therapeutic cargo that the needles could theoretically deliver are sensitive to extreme heat and cold. Natural and biocompatible materials that can achieve arrange of biophysical and mechanical properties following preparation in mild conditions are needed as drug delivery devices, and especially as raw materials for MN patches.

Silk fibroin (SF), derived from *Bombyx mori*, is a versatile, biocompatible, economical, and widely available material used in several FDA-approved medical devices.^22^ It offers unique advantages for MNs, including the ability to stabilize a wide range of therapeutic compounds through tunable chemical and physical properties. SF MNs can be chemically modified to optimize hydrophobicity, stiffness, and degradation rates, making them ideal for encapsulating and delivering therapeutics.^23^ Solid SF MNs can be loaded with cargo through coating, saturation, or incorporation during casting. MN-based delivery systems have been used in applications such as steroid contraception and vaccines,^15,16^ but their potential for delivering structurally sensitive proteins remains largely unexplored, especially compared to well-characterized hyaluronic acid and PLGA microneedles.^24,25^ Additionally, current SF MN fabrication techniques involve water vapor annealing, a process that requires temperatures above 35 °C and conditions that certain small molecules and peptides cannot survive during encapsulation.^26^ A final consideration when evaluating emerging MN delivery devices is the animal model used in testing; the vast majority of available literature has tested these devices in mice or rats, rather than a more appropriate analogue for human skin.

In this study, we demonstrate that SF MN patches can encapsulate and deliver AFPs into porcine skin without loss of function, using a fabrication process without hot or cold steps. Avoiding any heating during the encapsulation of the AFP cargo preserves protein structure and activity throughout manufacturing. The resulting patches readily degrade and deliver doses of AFPs that persist after patch removal. These findings establish SF MNs as a viable platform for transdermal delivery of AFPs and lay the groundwork for future therapeutic applications involving AFPs or other fragile proteins.

## Material and Methods

### Silk Solution Preparation

SF MNs were prepared based on established protocols,^16,21^ with modifications specific to AFP encapsulation and release (Figure S1A). In short, aqueous silk solutions were prepared from *B. mori* cocoons (Canon Inc., Tokyo, Japan) as described previously.^27^ Silk cocoons were cut into dime-sized pieces before boiling for 60 min in 0.023 M sodium carbonate solution to remove the sericin. The remaining degummed SF was removed from the solution and rinsed in deionized (DI) water. Water was changed every 20 min for a total of 3 times in 1 h. SF was removed from the water and placed on aluminum foil to air-dry for 24 h under continuous negative pressure. Dried fibers were dissolved in 9.3 M lithium bromide (LiBr) solution at 60 °C for 4 h. The dissolved SF was placed in 3.5 kDa molecular weight cutoff dialysis tubing (SpectraPor, Cole-Parmer, Vernon Hills, IL) and dialyzed in DI water to remove the salts. Water was changed 6 times over 48 h. The dialyzed solution was centrifuged twice at 9000 rpm for 20 min to remove particulates. Following concentration by air-drying and evaporation for 24 h, the resulting SF solution was approximately 12% weight/volume (w/v) and diluted to 7 or 10% w/v prior to casting in the molds.

### Microneedle Preparation

#### Mold Design

High resolution (17-μm x-y resolution) inverse molds were designed based on dimensions published prior^16^ and printed on a Form3B+ 3D stereolithography (SLA) printer (Formlabs Inc., Quincy, MA). Each mold contained a 20 × 20 array of conical needles that were 700 μm tall and 400 μm wide at the base, with a defined tip (Figure S1B,C). MNs were evenly spaced with 360 μm gaps between needles in a rectangular grid pattern on the patch base. Polydimethylsiloxane (PDMS) was used to make a flexible base mold for the patches, enabling nondestructive removal of the SF MNs from the molds.

#### Patch Fabrication and Raw Material Formulations

Prior to casting, all protein solutions were degassed in a vacuum chamber for 15 min to eliminate air bubbles that could interfere with molding. To concentrate AFPs within the MNs and adjacent patch base, two different SF solutions were prepared. In the first step, 500 μL of the AFP-loaded SF solution was centrifuged into the PDMS molds at 3000 rpm and 15 °C for 12 s. This ensured complete filling of the needle cavities and partial formation of the patch base. The centrifugation step was repeated 12 times, with a 90° rotation between each cycle to promote uniform distribution across the 20 × 20 MN array. Depending on the targeted AFP loading, 0–500 μg of fluorescein-labeled AFPs were incorporated into concentrated SF solutions and diluted to final concentrations of 7 or 10% (w/v) using DI water. In the second step, 1000 μL of non-AFP SF solution (7 or 10% w/v) was added to complete the patch backing, resulting in a total mold volume of approximately 1.5 mL. This volume was experimentally determined to be optimal for producing mechanically stable MN patches with needle dimensions within an acceptable margin of error (±10%) to those provided by the mold. Molds were dried in a fume hood under light-protected conditions for 48 h. The resulting patches (*n* = 101) measured 1.4 × 1.4 cm and contained 400 MNs, each measuring 620–660 μm in height with an average base diameter of 450 μm (Figure S2). All patches were stored in the dark at 25 °C, 4 °C, or −80 °C until use; no difference was observed in patch performance or AFP activity based on storage method.

### AFP-III Labeling

AFP-III was purified as previously described^10^ and dissolved at 10 mg/mL in 0.15 M NaHCO_3_ buffer (pH 8–9). NHS-fluorescein (5 mol equiv) was dissolved in N,N-dimethylformamide (1 mL per 5 mg NHS-fluorescein) and added to the protein solution. The mixture was stirred at room temperature for 3 h, then divided into four 3 kDa MWCO centrifuge filter tubes. Samples were washed with phosphate buffer by centrifugation (4300 rpm, 4 °C) for 1.5 h per cycle until ∼0.5 mL remained and flow-through absorbance reached ∼ 0.1 AU, typically after five cycles. Based on the final wash absorbance, residual unbound fluorescein was several orders of magnitude lower than the AFP concentration, indicating that free fluorescein did not significantly contribute to the fluorescence signal measured in the release experiments.

### SEM Imaging

Microneedle samples were mounted on SEM studs using carbon tape and sputter-coated with gold for 120 s using an Emitech SC7620 mini Sputter Coater (Quorum Technologies). Imaging was performed the same day on a Zeiss EVO MA10 SEM (Zeiss) at an accelerating voltage of 5 kV.

### Mechanical Testing

Quartered SF patches (10 × 10 needles, ∼49 mm^2^) were trimmed to remove sidewalls, leaving only the flat base and needle array. This ensured a uniform surface for even force application across needle tips. Compression testing was performed on quartered SF MN patches using a CellScale Univert Mechanical Test System (5 kg load cell). A 0.1 N preload was used to ensure full contact with the platen. Patches were compressed to ∼35–40% strain at 1% strain/s, with a maximum load of 50 N. The modulus of elasticity was calculated from the linear region of force–displacement curves using CellScale software and measured specimen dimensions.

### Encapsulated AFP Quantification

The AFP content in the SF MNs was quantified using a fluorimeter (G1103B Cary 8454 UV–vis Spectrophotometer, Agilent) at 25 °C. Initially, a calibration curve was generated from known AFP concentrations based on labeled AFPs, as described above. Sectioned SF MNs loaded with various AFP amounts (0, 50, 250, and 500 μg) and weighing an average of 90 mg were then partially dissolved, meaning the AFP-containing needles were dissolved but the silk-only backing was preserved, over 120 min (reaching ∼30% dissolution by weight, indicating needle portion of patch was liquefied, as quantified by weighing patch before and after) in 2 mL of DI water. Fluorescence was quantified based on the calibration curve.

### Nanoliter Cryoscopy

Thermal hysteresis activity was determined in pure water using a Clifton Nanoliter Osmometer. Experiments were performed with a cooling rate of 0.075 °C/min and without annealing steps. Measurements were performed multiple times and on independent samples, following protocols and guidelines established elsewhere.^10,28^

### Porcine Skin Testing

Porcine skin was obtained from Northwest Premium Meats (Nampa, ID). Hog ears (*n* = 6) were washed with phosphate buffered saline, sanitized with iodine surgical scrub, dried, and shaved. Full-thickness skin sections were removed from the cartilage using a scalpel and tweezers. MN patches were inserted into thawed porcine skin using a 3D-printed screw clamp (Figure S4) to ensure an initial penetration depth of ∼350 μm and set in place for 60 min at 21 °C. Following removal, skin samples were fixed in 4% paraformaldehyde at room temperature for 1 h and stored at 4 °C. FITC-labeled AFPs were incorporated into the silk solutions prior to mold casting. Following complete drying of the MN patches, a patch was applied to sectioned porcine ear skin using 3D-printed screw clamps for 30–60 min to facilitate AFP transfer. Treated skin samples were subsequently frozen in optimal cutting temperature (OCT) medium, cryo-sectioned at 45 μm thickness using a Leica CM1950 cryostat, and mounted onto glass slides. Fluorescence imaging was performed using a Zeiss LSM900 confocal microscope.

### Statistical Analysis

All data are presented as mean ± standard deviation unless otherwise specified. Statistical analyses were performed using Prism 9 (GraphPad Software, La Jolla, CA). One-way analysis of variance (ANOVA) followed by Tukey’s post hoc test was used for multiple group comparisons. Statistical significance was defined as *p* < 0.05, with *p < 0.05, **p < 0.01, and *p < 0.001. Mechanical testing data sets were evaluated using unpaired two-tailed *t* tests with Mann–Whitney correction.

## Results

### Heat-Free Manufacturing Largely Preserved Patch Dimensions and Retained Patch Quality

Heat-free fabrication methods of 7 and 10% SF MN patches resulted in patches that effectively encapsulated AFPs. Figure 1 shows the morphology of AFP-loaded SF MN patches. AFP encapsulation visibly changed the MN color from clear white to yellow due to the fluorescent labeling, providing a visual confirmation that encapsulation and equal AFP distribution were achieved. The average height of 640 μm and base diameter of 450 μm confirmed that the MN dimensions varied slightly from the dimensions of the original stereolithographic resin base molds, with a reduction in MN height of ∼60 μm and a widening of the base diameter by ∼50 μm (<10% difference; Figures S2 and S5). This divergence is due the loss of resolution during the negative PDMS mold translation and the silk solution pulling away from the PDMS needle imprints during the drying process. Notably the change in needle dimensions did not alter needle functionality or formation and the penetration of porcine skin was not affected.

**Figure 1:**
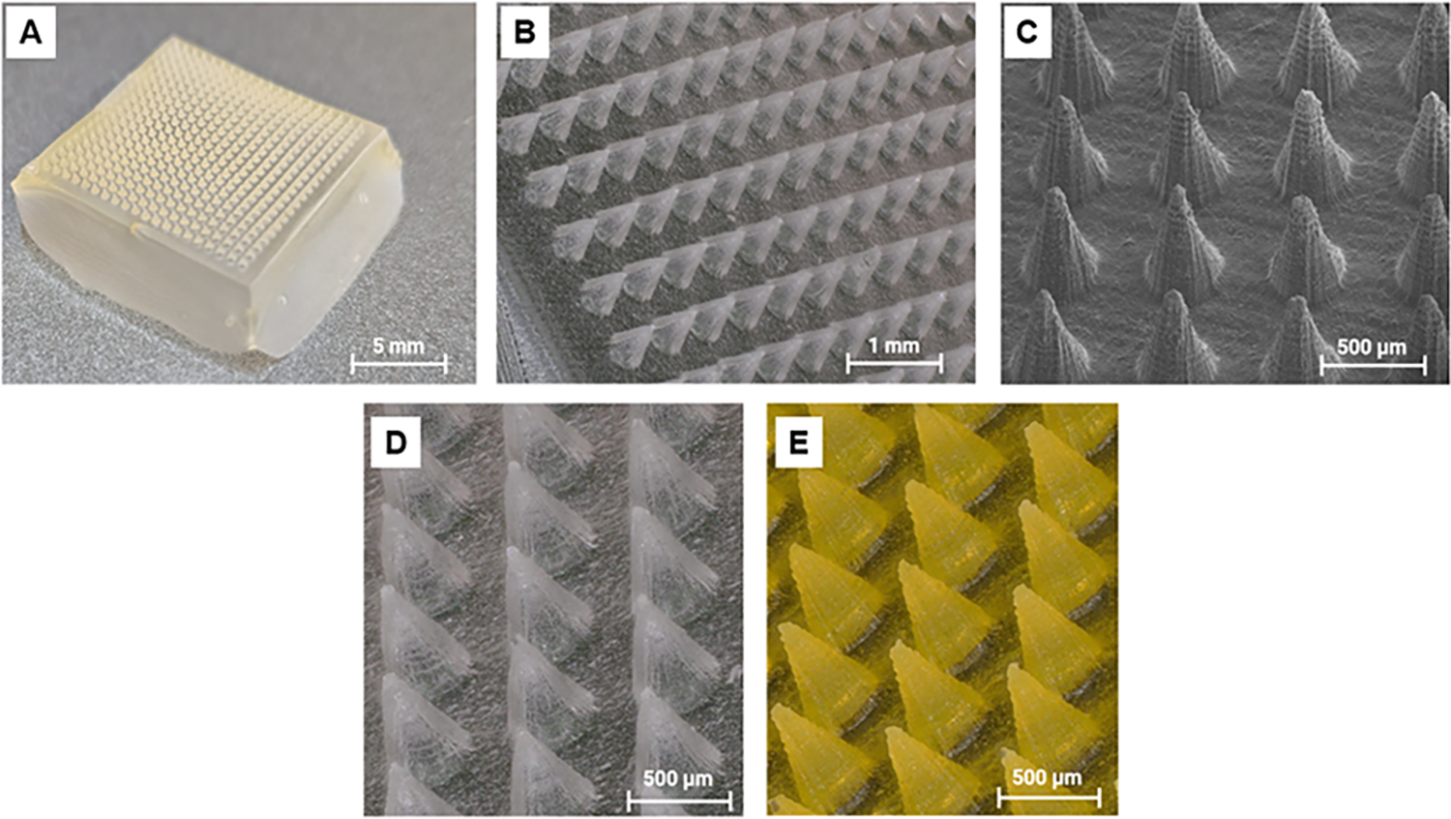
Morphology and of SF microneedle (MN) patches. (A) Optical image (1×) of a 50 μg AFP-loaded SF MN patch.(B) Digital microscopy image (4×) showing a uniform needle structure. (C) SEM image (100×) highlighting tip sharpness and regular array geometry. (D) Digital microscopy image of an unloaded control and (E) 500 μg AFP-loaded SF MN patch. Note the obvious yellow-green color in the tagged AFPs encapsulated within the patch in (E). Scale bars as shown in each panel.

#### SF MNs Encapsulate and Release Functional AFPs

SF MN patches were fabricated with varying amounts of AFP type III (0, 100, 250, and 500 μg) using 7 and 10% (w/v) SF formulations. Fluorescence spectrophotometry confirmed efficient encapsulation across the full range of AFP loads (10–500 μg), corresponding to over 0.1% (w/v) relative to SF. Incorporation of AFPs, including GFP-tagged variants, did not affect patch formation, needle morphology, array geometry, or mechanical properties (Figure 3C). AFP-loaded patches exhibited a visible color change but retained the same physical characteristics as unloaded patches, even after extended storage at both 25 and −80 °C (Figure 1D,E). We found that in DI water, 50% of the AFP payload was released within 15 min, and 70% within 400 min, as shown in Figure 2A. The patches further exhibited an average mass loss of 32.0 ± 1.0%, with visible transfer of AFPs from the microneedle tips into solution following 2 h in DI water. Patch dissolution was not influenced by variations in the drying process and occurred readily upon exposure to hydrated porcine dermal tissue or aqueous solutions. Figure 2B shows that dissolved AFPs released from SF MN patches exhibit thermal hysteresis activity of ∼0.3 °C comparable to native AFP III at similar concentrations, as measured by nanoliter cryoscopy. These results indicate that encapsulation and release from the MN patches preserve the antifreeze activity of AFP-III.

**Figure 2:**
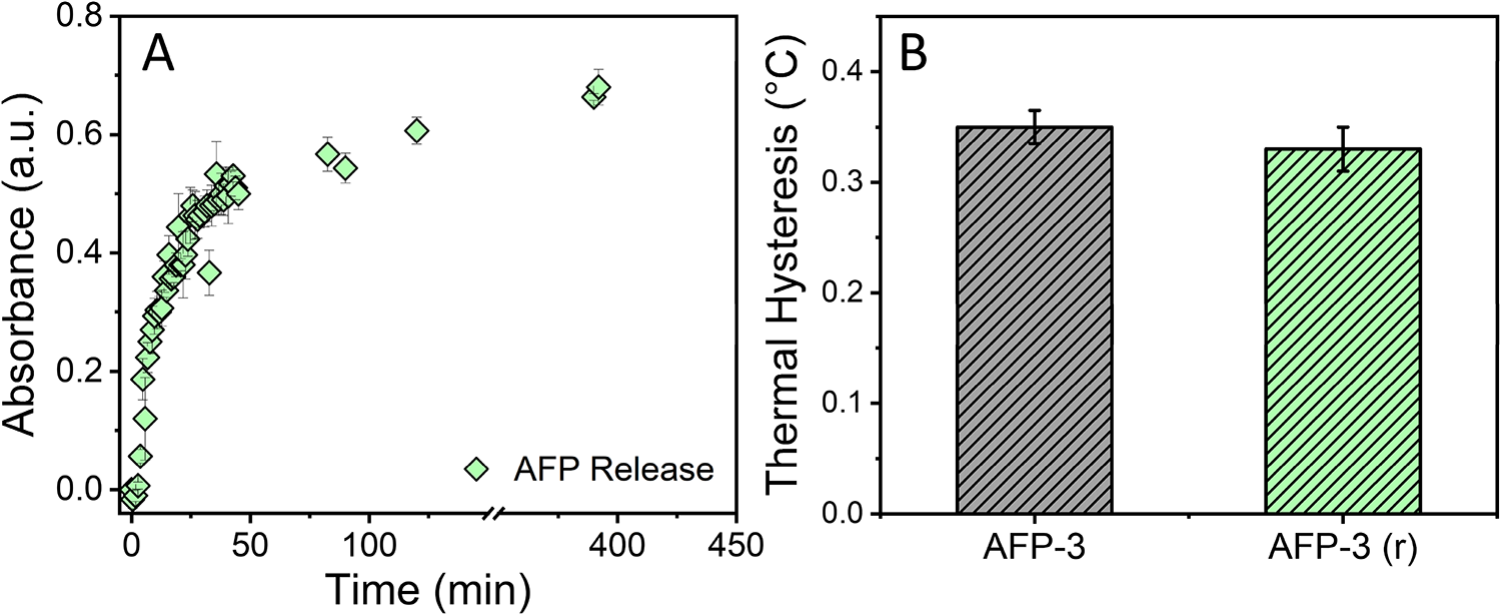
Release profile and functional integrity of AFP-III from 7% silk SF MN patches. (A) Fluorescence measurements from patches dissolved in water indicate that approximately 50% of AFP content is released within 15 min, with release reaching ∼70% by 400 min. (B) Thermal hysteresis measurements show that AFPs retain antifreeze activity recovery from dissolved SF MN patches.

### SF MNs Effectively Penetrate Porcine Epidermis

Both AFP-loaded and unloaded SF MN patches demonstrated sufficient mechanical strength to penetrate porcine skin (Figure 3), marking, to our knowledge, the first successful demonstration of a human-analog skin insertion using silk-based MN arrays. Uniaxial tensile and compressive testing revealed that the mechanical properties of the patches were strongly influenced by silk fibroin concentration, with 7 and 10% SF formulations exhibiting average elastic moduli of 6.67 and 7.55 MPa, respectively (Figure 3C). The inclusion of AFPs did not significantly alter the stiffness or ultimate tensile strength of the patches (Figure 3C). These mechanical characteristics enabled consistent insertion of the MN arrays into the upper epidermal layer to depths ranging from 100 to 300 μm. For comparative reference, the mechanical properties of human epidermis were obtained from published data^18^, confirming that the SF MNs possess adequate stiffness for transdermal penetration and delivery in human tissue. Postcompression analysis showed 40–100 μm reductions in needle height, with >75% of needles exhibiting tip blunting or tip breakage (Figure S3). A Student’s *t* test comparison of the mechanical elastic modulus for the 7% and 10% SF MN patches with and without AFP encapsulation showed no significant differences between 7% loaded and unloaded MN patches (*p* = 0.34), 10% loaded and unloaded MN patches (*p* = 0.28), or 7% and 10% unloaded (*p* = 0.1) and loaded (*p* = 0.09) patches.

**Figure 3:**
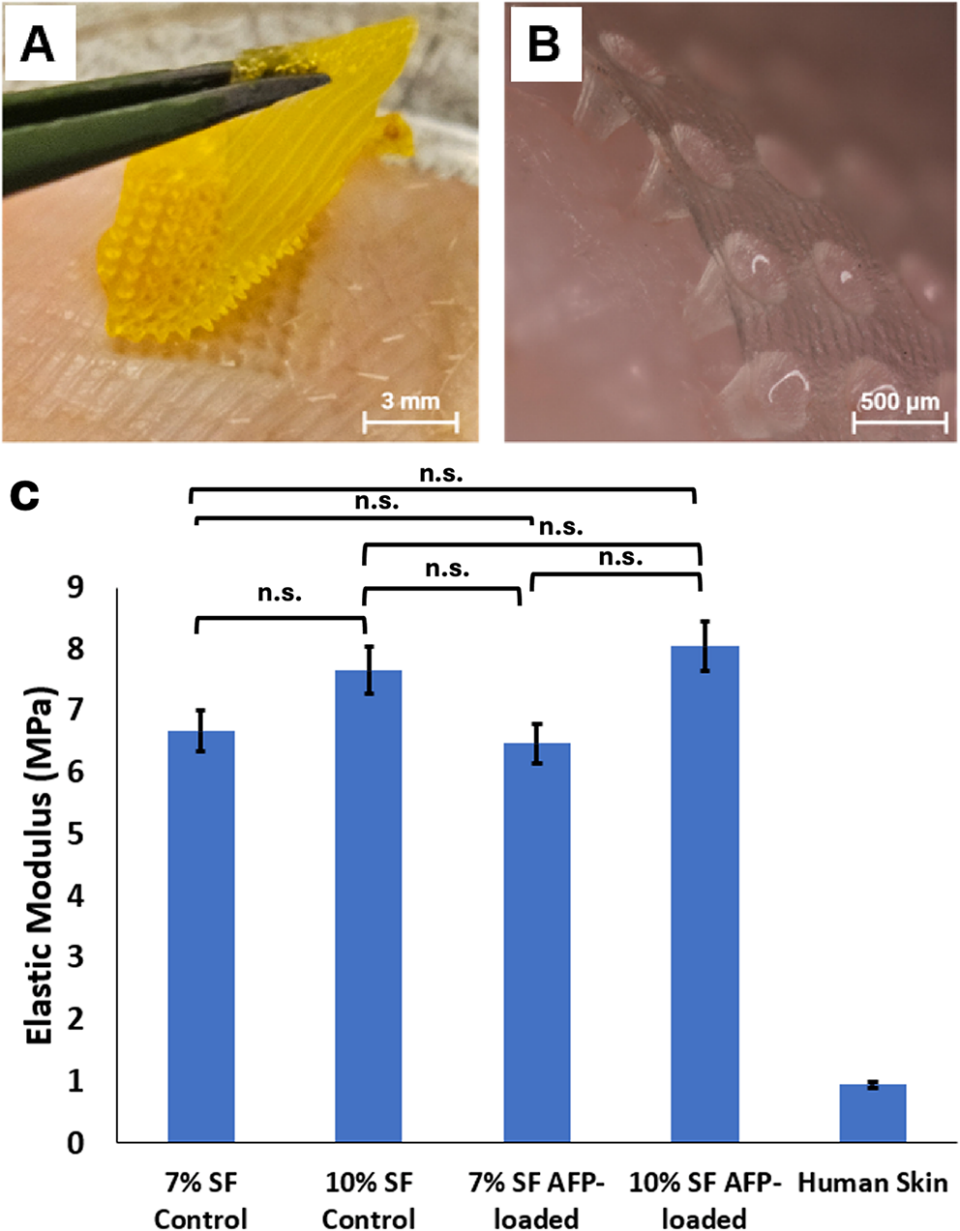
Epidermal penetration and mechanical properties of SF MN patches. (A) Optical image of a quartered 500 μg AFP-loaded SF MN patch following compression into porcine skin. (B) Image of a nonloaded SF MN patch inserted ∼300 μm into porcine skin without active compression, demonstrating that the needles readily penetrate the outer epidermis. (C) Comparison of mechanical stiffness (elastic modulus) for 7 and 10% SF MN patches with and without AFP encapsulation relative to reported values of human skin.^18^ No significant differences were identified between 7% loaded and unloaded MN patches (*p* = 0.34), 10% loaded and unloaded MN patches (*p* = 0.28), or 7 and 10% unloaded (*p* = 0.1) and loaded (*p* = 0.09) patches. Average human skin elastic modulus from the literature is provided for visual comparison only and was not statistically evaluated against the MN patch mechanical data. Columns represent mean ± standard deviation. Photos taken by authors. n.s. = not significant.

### AFPs Are Effectively Delivered Into Dermal Tissue

To evaluate the delivery efficiency of AFPs from SF MN patches, fluorescently labeled AFP-loaded and control (unloaded) patches were applied to *ex vivo* porcine skin and left in place for 30 min. During this period, a substantial portion of the patch and needles dissolved and facilitated cargo release. The strong fluorescence signal in the epidermis following the application of AFP-loaded MNs (Figure 4) confirms successful deposition of AFPs into tissue. Fluorescence was not only observed at the expected depths corresponding to our needle height, but also deeper within the skin, suggesting diffusion beyond the original insertion zone.

**Figure 4:**
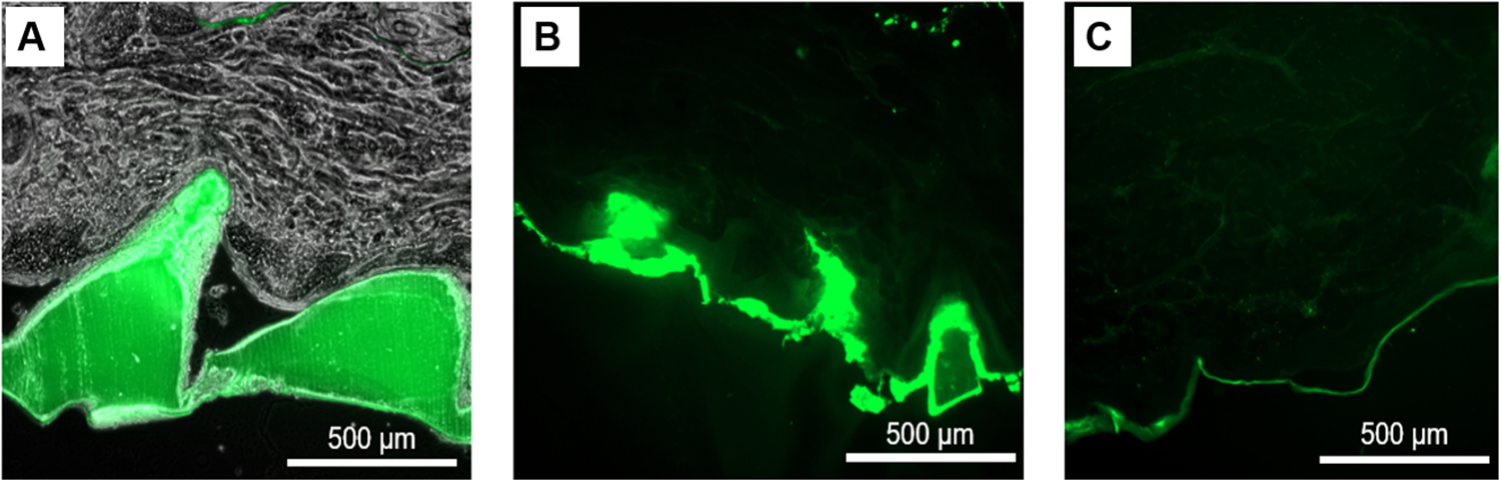
SF MN patches effectively deliver AFPs into porcine skin. (A) Histological image of cryo-sectioned porcine skin with embedded AFP-loaded (7% SF, 500 mg AFP) microneedles shows that fluorescence remains confined to the microneedles immediately after insertion. (B) After a 30 min application and subsequent removal of the AFP-loaded MN patch, with significant patch dissolution, fluorescence is observed throughout the epidermis and extends beyond the original needle positions, confirming release and dispersion of AFPs into the tissue. (C) Skin treated with a non-AFP-loaded (control) MN patch for 30 min under similar conditions shows minimal fluorescence. This indicates that the signal in panel (B) is attributable to AFPs and not to silk matrix or tissue autofluorescence. Images at 4×; scale bars = 500 μm.

## Discussion

We developed a fabrication method to produce silk SF MN patches that are mechanically robust and compatible with sensitive therapeutic cargo, in this case AFPs. These patches are prepared without heat, chemical cross-linkers, or postprocessing steps, and are capable of penetrating porcine skin, which has mechanical properties similar to human skin.^29^ The system supports encapsulation of biologically active biomolecules like AFPs, and enables transdermal delivery while preserving activity. Mechanical strength of the SF MNs is achieved through crystallization of silk during ambient drying, without requiring thermal or chemical treatment. Notably, the mechanical strength allowed the MN patches to pierce tough porcine skin prior to dissolving and releasing fluorescently labeled AFPs into the tissue.

An advantage of the MN formulation used in this study is the rapid dissolution and cargo release time. The needles dissolved within 30 min to 2 h, and over 70% of the encapsulated AFP was released within 1 h when tested in water (Figure 2A). The AFPs retained activity (Figure 2B) even after prolonged storage in the MNs at cold temperatures, suggesting the silk preserved the AFP activity, which was also not lost during the encapsulation and casting process. Inclusion of AFPs in the silk mixture did not impact patch mechanical properties.

Patches prepared with 7 and 10% w/v SF concentrations exhibited average elastic moduli of 6.67 and 7.55 MPa, respectively, values sufficient to penetrate human skin without needle fracture (Figure 3c) and comparable to those achieved using other cross-linking methods.^30^ In contrast, previously reported lyophilized MNs typically exhibit moduli in the range of 200 to 350 kPa, which limits their use to softer murine skin.^31,32^ Most MNs tested in mice have the same dimensions as those used in our study,^16,21^ suggesting that the penetration extends beyond the murine transdermal anatomy^33^ when the needle lengths exceed 600 μm. This may occur in humans, however the human epidermal, dermal, and subdermal layers, as well as adipose tissue distribution, necessitate the relatively long needles. Porcine skin anatomy more closely mirrors human layer thickness and toughness, indicating that the AFP diffusion observed in the *ex vivo* experiments could likely occur in human tissue as well. Further, the signal intensity scaled with AFP concentration and demonstrated dose-dependent delivery, with greater fluorescence seen in patches delivering 500 mg AFP versus 100 mg AFP (data not shown). These findings indicate that AFPs remain stable and functional following encapsulation, storage, and release from SF MN patches, and can be effectively delivered into deeper-laying dermal tissue. To our knowledge, our patches are the first to show effective penetration (Figures 3A,3B, and 4A) and cargo distribution (Figure 4B) in porcine skin, a more appropriate human analogue.

A significant and still-underexplored advantage of SF MN patches is their modularity and potential to be chemically and mechanically tuned for diverse applications. Our findings suggest that use of SF MNs in living tissues, a planned next step, will result in effective AFP distribution throughout the epidermis, as local circulation will move AFPs away from the original patch application site to a greater degree than what we observed in the *ex vivo* tissues. Relatedly, as MNs do not rely on systemic drug distribution, they potentially overcome a significant drawback of oral or intravenous drug delivery. Local drug distribution from the patches may minimize side effects and reduce doses needed to achieve bioactivity, resulting in increased patient compliance and lower manufacturing and prescription costs. Additionally, the SF from which the patches are made is hydrophobic, meaning it can stabilize numerous types of therapeutic compounds and prevent breakdown from ambient moisture during storage. Conversely, the SF patches can have increased hydrophilicity if desired, by incorporating biocompatible additives such as hyaluronic acid into the silk solution.^34–36^ Finally, the drying methods in this study were optimized to tune the mechanical properties of the MNs toward matching generalized human skin, but could feasibly be altered to produce patches that can mimic and penetrate the tough skin of the hands and feet, or be made softer to reduce tissue damage when delivering medications to more sensitive skin, such as that found in the axilla or the face.

Although this work focuses on AFPs, the fabrication approach is likely applicable to a broader range of peptides and protein therapeutics. Unlike MNs reinforced with additives such as glutaraldehyde,^37–39^ which can impair bioactivity and raise biocompatibility concerns, the used formulation only uses SF and the therapeutic cargo. This reduces the risk of denaturation and avoids introducing potentially toxic substances. The method also avoids water vapor or heat annealing, relying instead on β-sheet formation during drying to provide structural stability. Harnessing silk’s natural crystallization enables encapsulation of thermally sensitive cargo, including biologics that cannot tolerate conventional processing conditions, including AFPs. While we chose to store the patches at −25 and −80 °C to assess AFP longevity, the MNs are shelf-stable at room temperature (data not shown; see Figure 1A,B for patch in ambient conditions), meaning stable cargo could also be prepared and stored without the need for harsh hot or cold conditions.

Finally, delivery of AFPs into porcine skin using SF MNs lays the groundwork for potential future applications for preventing cold-related injuries. Future directions will evaluate if transdermal administration of AFPs can reduce the severity of frostbite in remote or extreme environments. Such a system may support cold-storage of complex tissues like organs by rapidly delivering defined quantities of AFPs to the desired organ layers, potentially extending viability and increasing organ availability. An estimated 17 people die in the U.S. each day waiting for a transplant organ, with up to two-thirds of potential organs being discarded due to long transport times and short storage viability.^40^ Microneedles may be an effective solution for preserving transplant organs for longer timeframes at lower temperatures, as they could eliminate the need to expose the entire organ to cryoprotectants, and instead target delivery to the most cold-susceptible regions. More research is needed to determine if the patches can prevent cold damage and how targeted and localized AFP delivery can be optimized, perhaps in combination with pulsatile flow bioreactors, to preserve organs and tissues in subfreezing temperatures.

This proof-of-concept study focused exclusively on AFPs and thus had several limitations. Additional studies are needed to test the compatibility of this MN fabrication process with other biologics. *In viv*o evaluation of distribution and functional activity following delivery is also required and is planned for future work in small and large animal models. Cold protection in tissue was also not assessed in this work and is the subject of ongoing investigation. Future efforts will address tuning dissolution profiles for different applications. While SF MNs can be engineered for extended release, rapid delivery was appropriate for cryopreservation-focused use cases, where rapid delivery of AFPs is warranted. This study also evaluated patch dissolution and release in DI water, rather than PBS, as we did not include live cells or tissue viability end points, which will be the subject of future work carried out in physiologically relevant buffers. Finally, this study did not quantitatively evaluate how much AFP was delivered to the tissue, instead focusing on “proof of concept” delivery of active AFP. Though future work will address this limitation, there is no literature that defines precise quantities of AFPs needed for tissue preservation. An additional challenge is that, once delivered to the tissue, AFP would break down in any recovery process necessary to precisely quantify it. For these reasons, we did not quantify the exact amount of AFP in the porcine skin in the present study beyond the visible fluorescence. Based on the MN dissolution data (Figure 2A) it is likely that a similar amount of AFP was transferred to the porcine tissue, as the natural tissue hydration during the application process dissolved the patches. Future work will quantify precise AFP amounts.

## Conclusion

SF MNs offer a versatile platform for the transdermal delivery of emerging therapeutics, including temperature-sensitive biomolecules. The present work demonstrates that SF MNs achieve reliable insertion into porcine epidermis, a mechanically relevant analogue for human skin, and rapidly release encapsulated AFPs without requiring chemical cross-linkers or thermal processing. This fabrication approach preserves protein bioactivity while minimizing safety concerns associated with conventional reinforcement methods. AFP-loaded SF MNs show promise as future cryoprotectant delivery devices that may support extended cold storage of donor tissues and organs. In addition, SF MNs may potentially be deployed for localized protection against cold-induced tissue injury or adapted for the delivery of other labile peptide therapeutics. These findings highlight the feasibility of using SF MNs for a range of biomedical applications and support their further development as a broadly applicable delivery system for fragile therapeutics.

## Supplementary Material



## Notes

Additional details regarding the microneedle fabrication and characterization processes; schematic of the standard microneedle casting process (Figure S1); imaging to highlight the detail of the needle tips once they are removed from the mold (Figure S2); microscopy showing how the needles deform but do not break under forces far greater than what is required to pierce human skin (Figure S3); graphical representation of the custom 3D printed screw clamp used to ensure the needles are inserted into porcine skin with uniform and repeatable force (Figure S4); and schematic highlighting the unique steps in the microneedle fabrication method that allow it to be completed at room temperature (Figure S5) (PDF)
