# Supplementary material for: Silk Microneedles for Targeted Epidermal Delivery of Antifreeze Proteins
Source: ACS Omega. 2026 Mar 16;11(12):19023–30. doi: 10.1021/acsomega.5c11565 (PMC13044823; doi:10.1021/acsomega.5c11565)
Supplement: Supplementary file 1 [file ao5c11565_si_001.pdf]

Supporting Information Cover Page

Silk Microneedles for Targeted Epidermal Delivery of Antifreeze Proteins

Brian T. Penney[1*], Antonio Reyes[2*], Calvin Jones1, Jordan Daw[3], Amevi Semodji[1], Konrad Meister[2,4#], Sophia K. Theodossiou[3,5#]

[1]Biomedical Engineering Doctoral Program, Boise State University, Boise, ID 83725, USA.
[2]Department of Chemistry and Biochemistry, Boise State University, Boise, ID 83725, USA
[3]Department of Mechanical and Biomedical Engineering, Boise State University, 1910 University Dr MS2085, Boise, ID 83725, USA
[4]Max Planck Institute for Polymer Research, 55128 Mainz, Germany
[5]Spencer Fox Eccles School of Medicine, University of Utah, Salt Lake City, Utah 84113, USA

*equal contribution
#Shared corresponding authorship. Address correspondence to sophiatheodossiou@boisestate.edu and konradmeister@boisestate.edu
Keywords: Microneedles, silk fibroin, antifreeze proteins, frostbite, porcine skin

Supplemental Figures

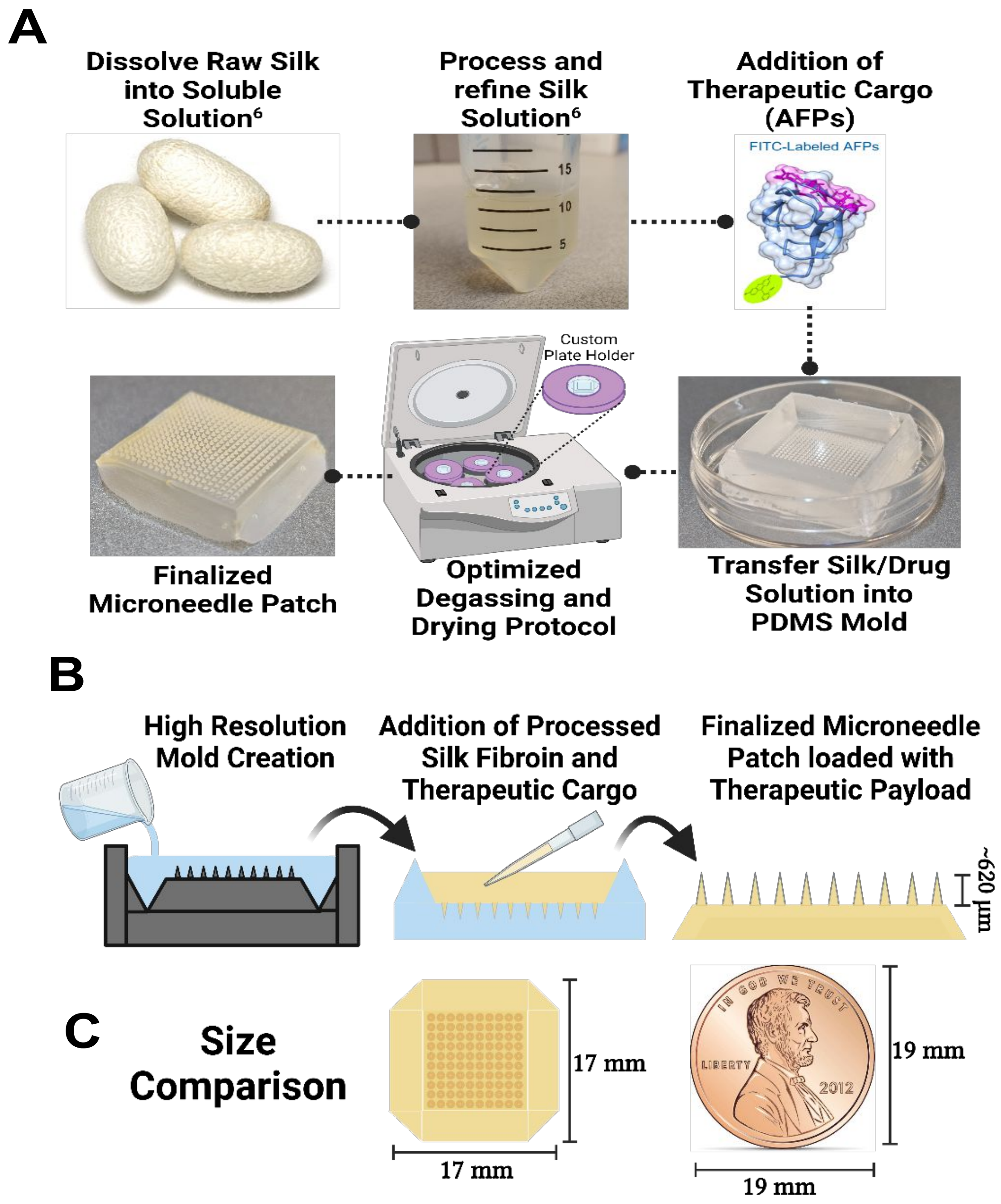

Figure S1. (A) Schematic overview of MN fabrication. Silk cocoons from the B. mori silkworm are boiled, solubilized, dialyzed, and diluted to 7 or 10% w/v solutions, which are then cast into PDMS molds of the microneedle array. (B) Casting process details. SF Solution containing 500 mmol/L AFP was cast into the custom PDMS mold, solution is air dried so β-sheeting can occur, and mold is removed and stored at room temperature or frozen. (C) Resulting MN patches have dimensions of 17 x 17 mm, approximately the size of a penny.

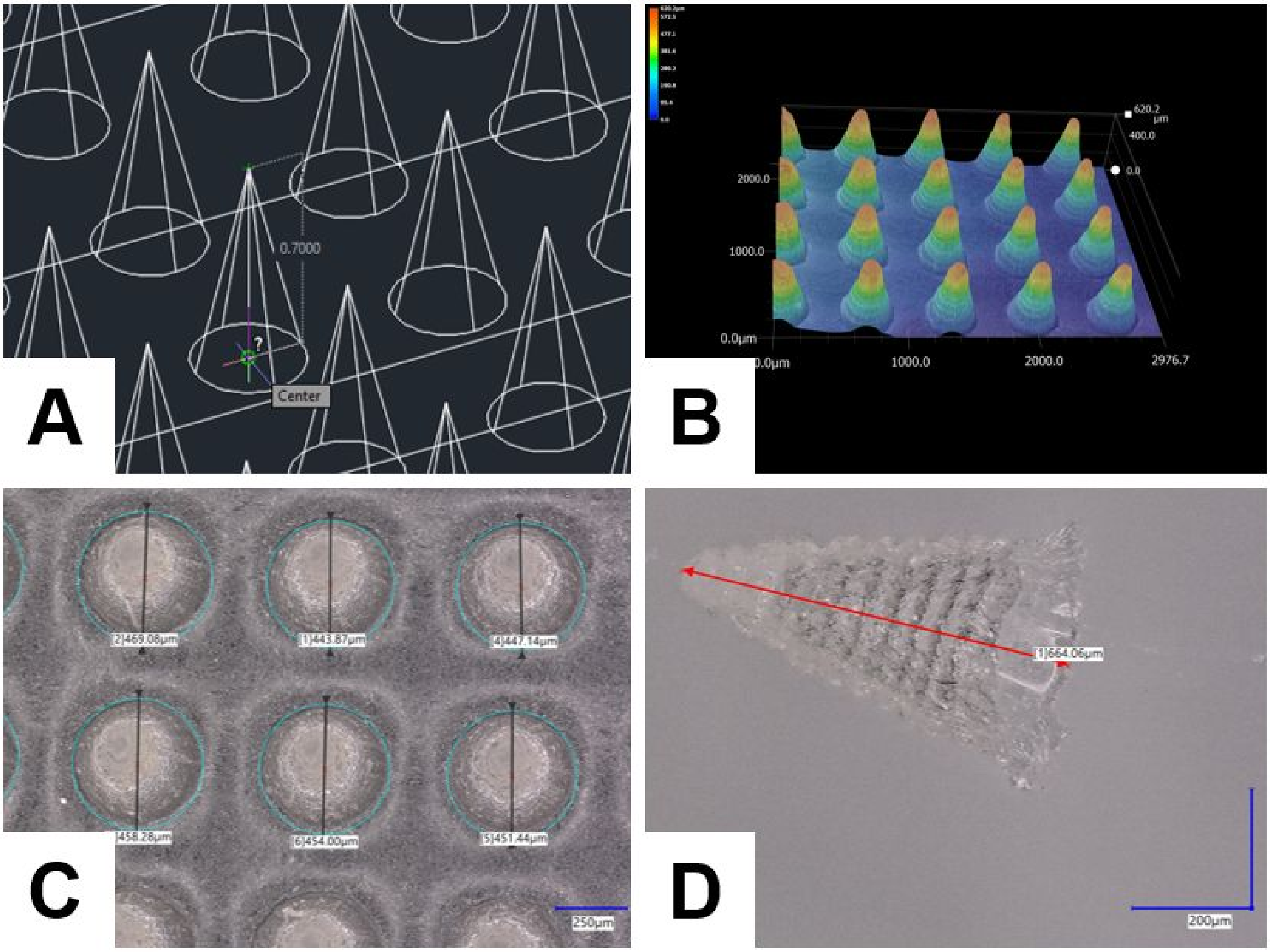

Figure S2. Dried SF MN patch needle height and base width (B, C, D) differed from the AutoCAD (A) designed heights (700 microns tall and 400 micron base width), the dimensions chosen based on previous work[20]. Average SF needle base diameters were 450 µm with average heights ranging from 620 - 660 µm. This disparity was due to the SF MN patch pulling away from the PDMS mold during the drying stage as well as lower resolution PDMS molds compared to the original resin printed molds resulting in wider bases and shorter needle heights. This change in dimensions can be anticipated throughout various mold-based microneedle fabrication processes and does not affect MN performance or function. Scale bars: (C) 250 µm and (D) 200 µm.

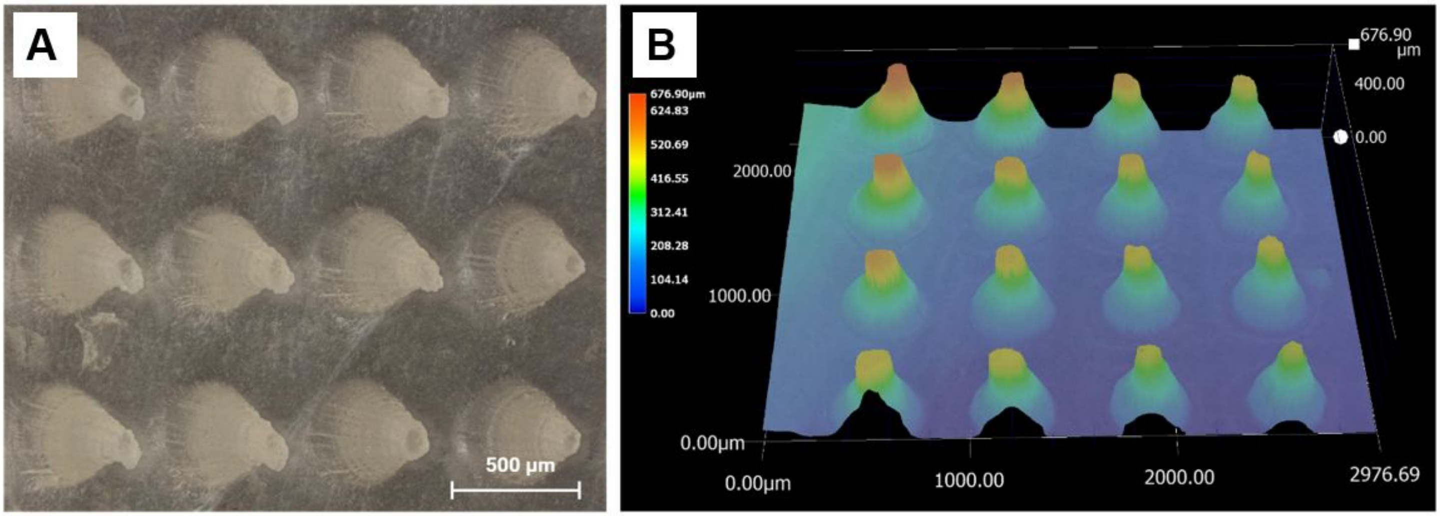

Figure S3. MN tip failure from compression testing. (A) Digital Image of 7% SF MN post compression testing, showing tip failure occurs at several MPa beyond the maximum penetrative stress required to pierce human skin. Though the tips break, the patch remains intact as long as conditions are dry. (B) Digital 3D depth compositional rendering of broken MN tips post compression testing was used to evaluate patch condition following compression testing.

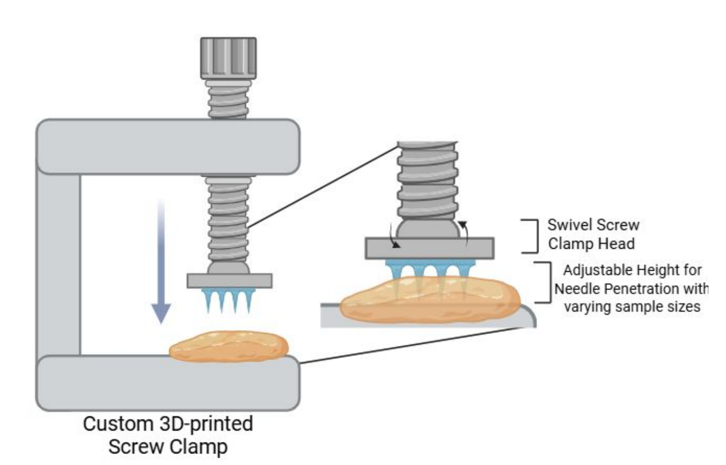

Figure S4. Schematic of the patch testing setup. The MN patch is pressed into the skin by the clamp to ensure uniform force distribution over the square patch backing. A swivel head and screw ensure there was no torsion on the patch.

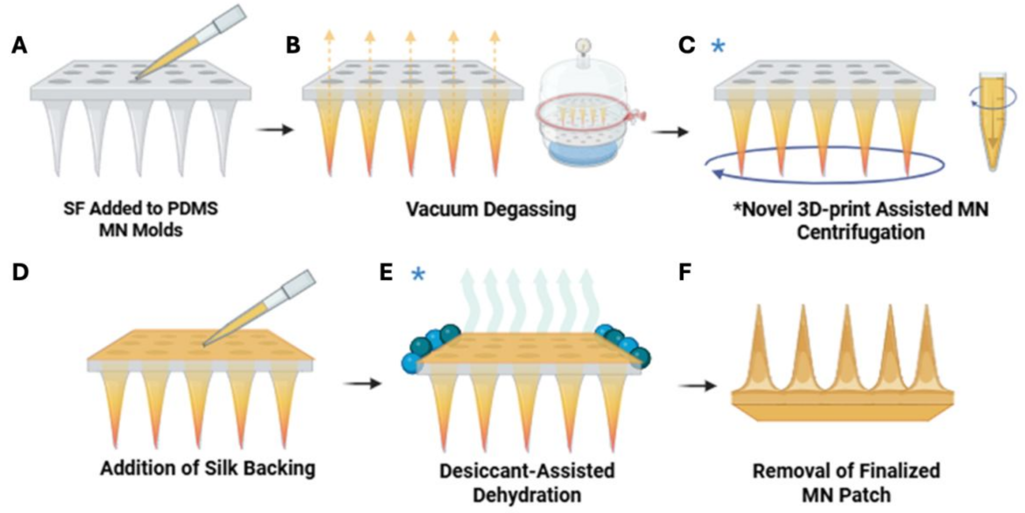

Figure S5**.** Schematic of the revised MN fabrication process. (A) Silk solutions are dispensed into PDMS MN molds then (B) degassed in a vacuum chamber for 15 minutes to remove bubbles from the solution. (C) Following degassing, filled molds are spun in a centrifuge utilizing a custom 3d-printed mount at 3000 rpm at 15° C for 12 seconds then rotated 90 degrees and repeated 11 additional times to finish with 3 full mold rotations. This process ensures the silk solution fully fills the MN shape in the PDMS mold removing trapped air pockets in the needle tips. (D) Additional silk is added to the mold to create a backing for the patch. Fully filled molds are then placed in a fume hood using light-protecting custom drying racks with (E) desiccant beads for 48 hours to fully evaporate the remaining water content in the solution. (F) Once fully dried, tweezers are used to remove the finalized MN patches. Asterisks indicate steps altered from previous protocols to ensure the process can proceed at room temperature.
